# Exploring what works, for whom, under what circumstances to transform systems: realist synthesis protocol of four ongoing studies and literature addressing health inequalities

**DOI:** 10.1136/bmjopen-2025-110455

**Published:** 2025-12-25

**Authors:** Stijn S Horck, Matty Crone, Carlijn B M Kamphuis, Gonneke W J M Stevens, Christine Dedding, Jet Bussemaker, Suzan van der Pas, Jantien van Berkel

**Affiliations:** 1 Department of Health Promotion, Maastricht University, Maastricht, the Netherlands; 2 Department of Public Health and Primary Care/Health Campus The Hague, Leiden University Medical Centre, Leiden, the Netherlands; 3 Department of Interdisciplinary Social Science: Public Health, Utrecht University, Utrecht, the Netherlands; 4 Department of Ethics, Law and Humanities, Amsterdam UMC, Amsterdam, the Netherlands; 5 The Institute of Public Administration, Leiden University, Leiden, the Netherlands; 6 Faculty of Social Work and Applied Psychology, Leiden University of Applied Sciences, Leiden, the Netherlands; 7 Chair Group Consumption and Healthy Lifestyles, Wageningen Universiteit and Research

**Keywords:** Health Equity, Health policy, Health Services

## Abstract

**Abstract:**

**Introduction:**

Health inequalities remain resistant to interventions that primarily target individual behaviour. Although systems approaches are increasingly promoted, their application in practice is often not well grounded in real-world settings. In this protocol paper, we present the approach we will take in an overarching project that synthesises the combined insights of four ongoing systems-based research projects on system-based approaches for reducing health inequalities in the Netherlands. By bringing together and comparing findings across diverse contexts, populations and interventions, we aim to generate an empirically grounded understanding of what works, for whom, in what contexts and why, and to derive actionable strategies for systemic change to reduce health inequalities.

**Methods and analysis:**

We use a realist approach to synthesise insights from the four ongoing projects. The design involves four iterative steps: (1) Identifying cross-cutting themes from project proposals and literature, (2) Developing and refining context–mechanism–outcome (CMO) configurations through literature review and Slow Science meetings, (3) Engaging Critical Friends to co-develop actionable strategies and (4) Assessing and validating these strategies across diverse contexts. Iterative feedback loops ensure continuous refinement, integration of stakeholder perspectives and exploration of emergent challenges. This design enables theory-informed, practice-based strategies to support sustainable system change in reducing health inequalities.

**Ethics and dissemination:**

Ethical approval for the four underlying projects has been obtained from the relevant institutional review boards, and the way their data is used for this overarching project falls within their approved scope. Dissemination will be ongoing and co-created with stakeholders, including policy briefs, factsheets, educational tools and academic publications, to support uptake of strategies for systems change.

**STRENGTHS AND LIMITATIONS OF THIS STUDY**

- This is the first overarching realist study to synthesise findings from four ongoing research projects, which enables theory-informed and practice-based learning across diverse contexts.
- The prospective design allows real-time engagement with empirical data, offering opportunities to refine and test strategies as they are being developed and implemented.
- Involving Critical Friends with lived, professional and scientific expertise ensures participatory validation and strengthens the practical relevance of findings.
- The diversity of target groups, interventions and theoretical approaches across the four projects increases the breadth of insights, but also poses challenges for synthesis and comparability.
- As the study depends on the progress and data availability of the underlying projects, variability in implementation or unforeseen delays may affect the scope of the overarching synthesis.

## Introduction

 Health inequalities have continued to rise due to complex interplays of social, economic and environmental factors.^1^ As a result, addressing these disparities has become an increasingly important priority on the policy agenda of the EU as well as various individual member states.^2 3^ Populations in vulnerable positions, such as those with low socioeconomic status, ethnic minorities and marginalised communities, tend to have significantly poorer health outcomes. Despite numerous research efforts and policy interventions aimed at reducing health inequalities, their impact has often been limited and served short-term results,^4^,^7^ and in some cases, they have even produced counterproductive effects.^8^

The study adopts a systems thinking perspective. Rather than referring to a single sector such as the healthcare system, systems are understood as the dynamic interplay of social, economic, political and institutional structures.^9^ From this perspective, health outcomes are seen not as the result of isolated individual choices, but as patterned behaviours shaped, framed and constrained by broader systemic conditions.^10^ Although systems approaches are increasingly promoted in health policy and research, their practical application often lacks sufficiently grounded approaches for operationalising systems thinking in complex, real-world settings.^11^ Therefore, many policies and interventions still focus on promoting individual behaviours (eg, ‘lifestyle drift’) rather than tackling the underlying systemic drivers. As a result, the structural barriers remain in place, hindering sustainable and equity-oriented system improvements.^12 13^

A major challenge is not merely recognising the need for systemic change, but actually changing the systems. This requires approaches that engage with the complexity of real-world settings and meaningfully involve the perspectives of affected stakeholders. We aim to explore how such change processes can be initiated and sustained, in order to reduce health inequalities in a more structural and lasting way. This requires a nuanced understanding of the contextual conditions, underlying mechanisms and stakeholder interactions that influence health outcomes across different levels of the system and how system changes may, in turn, reshape these dynamics.

To gain such understanding, we adopt a *realist approach* as a systems thinking perspective. This approach enables the examination of multiple system levels, their actors and their interrelations,^14^ including:

- **Micro-level** interactions between citizens (or patients or clients) and professionals/practitioners.
- **Meso-level** organisational dynamics, such as interorganisational collaboration and accountability structures.
- **Macro-level** considerations related to policy design and governance.

By employing this realist approach, our study aims to bridge the gap between theory and practice. It focuses on the development, synthesis and evaluation of insights from multiple research projects. In doing so, we seek to generate actionable strategies that support policymakers, practitioners and community stakeholders in shaping systems that promote health and respond effectively to the needs of vulnerable populations. What distinguishes this study from other studies that used the realist approach is its prospective design. Although Greenhalgh *et al*^15^ describe a similar realist approach, their ongoing engagement focuses on synthesising literature alongside active projects. In contrast, this study involves real-time engagement with empirical data, offering opportunities to adapt and further investigate the strategies and actions as they are being developed and implemented in practice.

### Study objective

The prospective study is an overarching project that examines common themes across multiple ongoing research projects that evaluate social programme interventions in diverse contexts and populations over a 4 year time period. By doing so, it enables an empirical exploration of how the initially identified programme theories hold up across entirely different settings. The purpose of this study protocol is to describe how we will conduct a realist synthesis of four systems-based research projects funded under the Dutch Research Agenda Program on Health Inequalities (2022). Each of these projects explores how systemic change can contribute to reducing health inequalities in populations in vulnerable positions (See table 1). With the synthesis we describe in this protocol, we aim to identify cross-cutting themes and mechanisms emerging from these projects, examine in what contexts they work and how, and derive actionable strategies for different system actors seeking to contribute to actual system transformation that reduces health inequalities.

**Table 1:** Overview of the four synthesised projects

Project	Countering syndemic vulnerability	Realist approach to social policies to realise health potential	Doing eHealth right	Minding the gap
General description	This project uses Participatory Action Research to co-create a tailored resilience approach in two cities, supporting individuals facing multiple health and social challenges. By addressing structural drivers of syndemic vulnerability, it aims to strengthen neighbourhood resilience through community engagement and policy-level action	This project uses a realist approach in Participatory Action Research to explore how and for whom social assistance and debt policies impact health, focusing on urban and rural municipalities in the Netherlands. Combining this with an institutional lens, it informs participatory research and futuring workshops to develop anticipatory strategies for healthier, more effective social policy systems	This project uses Participatory Action Research to investigate how to ensure that healthcare digitalisation does not worsen health inequalities for individuals with low socioeconomic positions. Collaborating with citizens and key stakeholders, it explores how structures, culture and practices can be transformed to keep healthcare accessible and inclusive	This project combines Participatory Action Research with complex adaptive systems theory to explore how poverty and mental health challenges interact in young people. Working in two neighbourhoods, it engages youth, parents, professionals and policymakers to co-develop systemic strategies that move beyond individual intervention
Study population	People with stacked health and social problems	Welfare benefit receivers and/or people with problematic debt	People with low digital access and key stakeholders of the digital health sector	Youth growing up in poverty aged 12–25 years
Systems approach	Community resilience approach	Realist approach and Institutional approach	Reflective system approach	Complex adaptive system approach

### Key research questions

To achieve this objective, the following five research questions will guide the synthesis:

1. What are key cross-cutting themes that emerge across the four projects in relation to addressing health inequalities through systems transformation?
2. Within these themes, what mechanisms are identified that explain how and why health inequalities are sustained or reduced?
3. What strategies and actions can influence, activate or disrupt these mechanisms to reduce health inequalities?
4. How do these mechanisms and strategies vary across different contexts, and what contextual factors enable or constrain their operation and effectiveness?
5. What actionable insights and strategies can be formulated for different system actors to guide their efforts to transform systems in ways that reduce health inequalities?

## Methods and analysis

### Rationale behind using realist synthesis

We chose the realist approach because its core principle—that social programmes and interventions produce different outcomes in different contexts depending on which mechanisms are triggered^15^—enables attention to the complexity of social policy interventions and systems, incorporates the perspectives of actors, and emphasises stakeholder engagement.^16^ These principles are essential for guiding the research process and informing the methodological choices outlined below. The study is planned to run from May 2024 to February 2028.

By evaluating the insights from these projects, the realist approach assures iterative reflection and refinement, helping to determine which lessons can be applied across different social programmes and contexts for real system changes. This makes the realist method particularly well suited for our study, as it accommodates the complexity of multiple intervention strategies implemented in diverse community settings, as well as the different research methods employed within the projects.^16^

For clarity, an overview of realist terms used in this protocol can be found in table 2.

**Table 2:** Definition of realist approach concepts

Concept	Description
Realist approach	A theory-driven evaluation method that seeks to understand how and why interventions or programmes work, or not, for whom, in what contexts and under which circumstances
CMO configurations	Context-mechanism-outcome (CMO) configurations are used to articulate and test hypotheses about how specific mechanisms are triggered in particular contexts to produce particular outcomes
Programme theory	A detailed explanation of how an intervention or programme is expected to work, outlining the causal pathways from actions or strategies to outcomes, informed by evidence and stakeholder assumptions
Grey literature	Non-peer-reviewed sources of information, such as policy documents, reports and working papers, that provide valuable contextual and practical insights

### Study design

**Figure 1:**
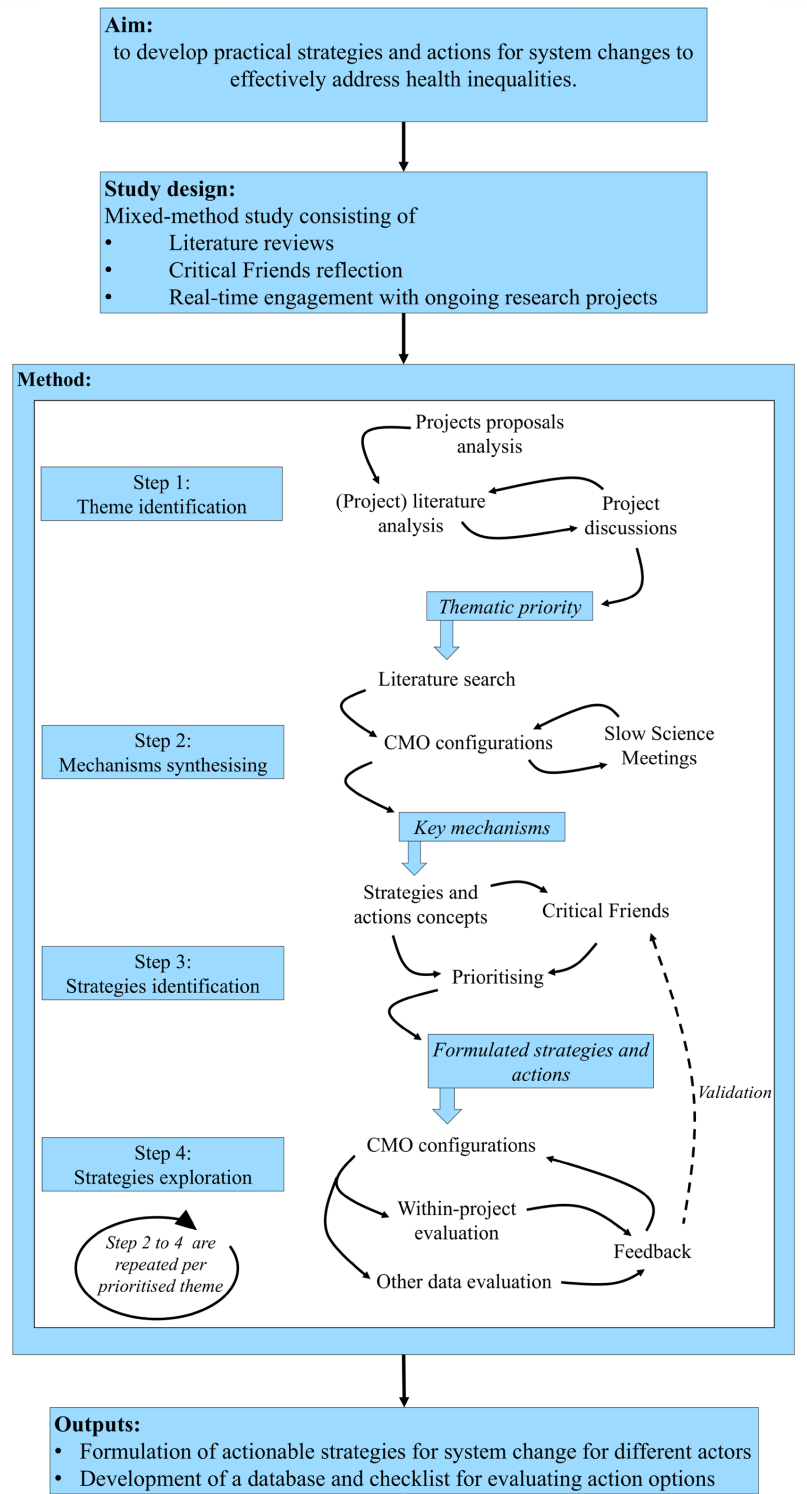
The study protocol.

The study design consists of literature reviews, reflections with Critical Friends (ie, stakeholders with experiential, professional and scientific knowledge, see step 3 for more detail) and real-time engagement with ongoing research projects (see figure 1). Developing theoretical insights and actionable strategies from the four projects involves multiple steps to synthesise findings. The continuation of the text will outline the consecutive steps we will undertake to answer the research questions and to achieve the objectives.

### Step 1: identifying cross-cutting themes shaping systemic drivers of health inequalities

To identify the most relevant themes that will guide the focus of this overarching project, the study starts with a comprehensive analysis of the project proposals and their referenced literature, combined with exploratory searches of additional literature, to develop a rough Initial Programme Theory (IPT) which is a preliminary explanation of how change is expected to occur and through which mechanisms, and under what conditions. First, this process evaluates how each project defines its core problem, considering relevant contextual factors and underlying mechanisms. Next, the anticipated outcomes are examined, including both the expected research findings and the practical system changes aimed to uncover to address health inequalities. This process will produce an overview of the aspects and elements that play a key role within each project, and how they relate to both the underlying causes of health inequalities and potential strategies to address them. Based on these insights, a preliminary codebook will be developed that will be used to analyse the literature that formed the basis of the theoretical and empirical foundations of each project proposal. This approach enables the identification of themes that may initially appear specific to one or two projects but are also reflected in the literature referenced by other projects. Therefore, identifying a broad range of themes that are, either explicitly or implicitly, embedded in the projects’ reasoning about the factors contributing to both the emergence and reduction of health inequalities. The underlying assumption is that these themes are therefore plausibly present in the eventual execution of the projects, even if they are not explicitly articulated in the project proposals. For clarity, the themes will be organised into groups and sub-groups to support further discussion and evaluation.

Based on the thematic overview, group discussions will be organised involving representatives from all four projects. These discussions will serve to further elaborate on the identified key themes and explore the specific aspects related to these themes. Together, the project teams will determine which themes should be prioritised for the next phase of the study. This prioritisation will be guided by several key considerations, including: (1) The feasibility of further investigation, based on an assessment of the likely availability of relevant data from the projects themselves, scientific and grey literature, or other practical examples, (2) The perceived urgency of the theme and the extent of existing knowledge gaps, particularly in terms of its potential impact, as informed by the expert judgement of the involved researchers and (3) Insights from interviews with stakeholders in the public health sector and beyond, to incorporate their perspectives on which themes identified so far warrant further investigation. The identified themes will be continuously reviewed and refined throughout the project to incorporate evolving insights and emerging priorities. This process will continue for as long as time and resources permit until the project’s completion.

### Step 2: synthesising the mechanisms behind the identified cross-cutting themes

Once a key theme is identified for further investigation, the next step involves formulating Context-Mechanism-Outcome (CMO) configurations which are the core analytical units in realist research, describing how particular contexts may trigger mechanisms that produce certain outcomes.^17^ This way, we will systematically examine and reflect on the interactions between context, mechanism and the intended outcome per theme. To achieve this, we will conduct a second, systematic literature review to develop and test initial programme theories using existing evidence. These theories, expressed as CMO-configurations, will then be iteratively refined by comparing them to patterns observed in the four ongoing projects and other empirical sources. This process involves revising, adding or reconfiguring elements of the CMO structure based on how well they explain the outcomes seen in practice.

The search strategy will follow an iterative approach fitting to a realist review.^18^ Once a theme has been prioritised, studies will be included when they offer explanatory insight into how contexts, mechanisms or strategies within that theme influence outcomes relevant to the IPT. Sources that only provide descriptive or correlational findings without explanatory contribution will be excluded. Unlike a systematic review, the aim is not exhaustiveness but to purposively identify evidence on the review themes that develops and refines CMO configurations, continuing until no new conceptual insights emerge. Searches will be conducted in PubMed, SCOPUS, PsycINFO, Web of Science and other databases identified by the research team. We will only include studies written in English and Dutch.

To enhance efficiency, we will use ASReview, an open-source machine learning tool that streamlines and prioritises literature screening, allowing for a more focused selection of articles for full-text appraisal to explore the CMOs.^19^ In line with the realist approach, grey literature will be used to inform, refine and assess the CMOs. Such literature often contains rich contextual descriptions of healthcare programmes, innovations and evaluations, making it a valuable source for exploring the mechanisms underlying the identified themes.^20^ We will purposively include grey literature that contributes to developing, refining or testing the IPT, particularly when it provides insights into how contexts, mechanisms or strategies operate that are not available in formal academic publications. While we will consider the methodological transparency and credibility of the sources, the primary criterion will be conceptual relevance for realist theory-building. As this consortium specifically investigates how Dutch systems maintain or reduce inequality, the relevant grey literature is predominantly Dutch and will therefore be included where conceptually relevant. As all consortium members are Dutch-speaking, these sources can be appraised without translation.

The CMOs will be further refined and validated through ongoing discussions in *slow science* meetings, involving researchers from the various projects. Slow science meetings are deliberately paced and thoughtfully organised gatherings that prioritise reflection and meaningful dialogue. They create space for participants to engage deeply with complex issues and encourage collaborative inquiry across disciplinary and institutional boundaries.^21 22^ These meetings will also help determine whether additional relevant literature needs to be incorporated, potentially looping back to the literature search phase for further exploration.

### Step 3: identify strategies and actions to influence mechanisms within and across different contexts

For this phase, this project makes use of Critical Friends. Critical Friends are stakeholders who, based on their scientific, professional expertise and/or personal lived experience, possess the knowledge and skills to ask critical questions that help assess and advance a situation.^23^,^25^ They include a diverse range of stakeholders such as people with lived experience, policymakers, insurers, advisory bodies, civil society organisations and individuals from the commercial sector. The selection of Critical Friends will be based on diversity in knowledge and experience, geographical spread and a shared commitment to learning and implementation. In addition to these criteria, we will purposively recruit individuals with varied professional backgrounds, lived experiences of inequity, policy-level influence and system-level implementation expertise. Diversity will be actively sought across dimensions such as sector, role and social identity where relevant to the topic.

The synthesised mechanisms central to one key theme are shared to foster meaningful reflections and discussions aimed at refining key concepts and shaping actionable strategies for system change. To facilitate engagement, annual sessions will be organised throughout the duration of the project, where the Critical Friends are asked to explore the identified mechanisms of a theme and contribute their perspectives. Depending on participant preferences, prior experiences and the nature of the topics, discussions will take place in both homogeneous and heterogeneous groups. Homogeneous groups will typically consist of participants with similar lived experience backgrounds, whereas heterogeneous groups will consist of combinations of policymakers, practitioners and researchers to support cross-perspective reflection. This approach ensures that different types of knowledge can contribute on equal footing, reducing the risk that professional expertise overshadows lived-experience expertise during co-reflection and decision-making. It creates structured opportunities for all participants to shape the development, interpretation and prioritisation of mechanisms and strategies, and helps prevent tokenistic involvement. Creative design-thinking strategies will be employed to accommodate diverse participation styles and encourage the articulation of emerging issues that may be difficult to express in traditional settings. These sessions will result in an overview of potential strategies and actions linked to specific mechanisms within a theme. This overview will then be discussed by the researchers involved in their projects, with priority given to strategies and actions that are considered most important for achieving sustainable system change, or that warrant further investigation because they may still hold unexplored potential.

Where possible, these strategies and actions will be investigated within the projects and complemented with evidence from grey literature and practice-based insights. For strategies not represented within the projects, the emphasis will lie on drawing from grey literature and insights from the projects’ shared networks. In keeping with the realist approach, CMOs will be developed for the selected strategies and actions, which can then be explored using data from the current projects and other relevant sources.

### Step 4: exploring formulated strategies and actions within different contexts

As the four projects progress, the developed actionable strategy concepts and their CMOs are assessed and evaluated within their respective contexts. This real-time evaluation will enable us to explore how well these strategies align with the practical realities of each project and to identify any necessary refinements.^26^ The assessments will draw on a combination of qualitative and quantitative data from the projects, including interviews, surveys, project reports and intervention outcomes. Additionally, new data will be collected through interviews with involved researchers to reflect on the CMOs and the strategies derived from them. This approach aims to provide a comprehensive understanding of their effectiveness and applicability. For strategies and actions that cannot be directly examined within the current projects, further evaluation will rely on data from other ongoing initiatives not directly involved with this study, case studies of other completed projects and relevant grey literature. As a final step, we will validate whether the strategies developed truly address the problems identified within the targeted themes, with a concluding review by our Critical Friends to capture the perspectives of those affected by the formulated strategies.

The findings from these evaluations will feed into iterative feedback loops, enabling the refinement of strategies, addressing unexpected challenges and potentially identifying the need to revisit and further explore other themes. Therefore, Steps 2 to 4 will be repeated throughout the project for each theme prioritised for deeper exploration, in order to develop actionable strategies for systemic change. The insights gained will be shared with the Critical Friends during the annual reflection meetings, ensuring that emerging lessons inform ongoing discussions and contribute to a shared understanding of effective system change approaches. This iterative process fosters continuous improvement and assessment of actionable strategies across the projects, strengthening the overall impact and relevance of them. This can be seen as an empirical approach to real-time piloting within realist synthesis, as described by.^15^ This synthesis will involve comparing insights across sources to generate explanatory patterns, exploring divergent findings to identify contextual factors or assumptions that may account for differences, and weighing evidence based on both rigour and relevance.^18^ By investigating strategies within specific contexts and consolidating findings across diverse data, we aim to produce practically grounded, context-sensitive explanations of which strategies and actions work for whom and under which circumstances.

### Ethics

This study protocol will be conducted in accordance with the principles of the Declaration of Helsinki. Ethical approval for the four underlying studies has been obtained from the relevant institutional review boards (approval numbers: WUR-REC 2024-157, The Medical Ethics Committee Leiden The Hague Delft 23-3102, Medisch Ethische Toetsingscommissie VUMC, 2022.0485, Ethics Committee of the Faculty of Social and Behavioural Sciences of Utrecht University 23-0414, 23-0194, 23-0330, 24-053). The use of data in the overarching project, as described in this protocol, falls within the scope of the ethical approvals granted for each of the four underlying studies.

## Discussion and dissemination

This protocol has notable strengths, including its prospective realist design, structured cross-project synthesis and participatory involvement of stakeholders, which supports theory refinement across real-world settings. However, certain limitations should be acknowledged. The synthesis depends on the progress, data availability and quality of the four underlying projects, which may differ in timing, methodological approaches and contextual focus. To mitigate this, we will supplement insights from the projects with grey literature, stakeholder consultation and iterative CMO testing across alternative sources where needed. Additionally, insights emerging from specific contexts may not be directly transferable to other settings. To address this, the analysis will prioritise explanatory patterns rather than universal claims, highlighting contextual contingencies and boundary conditions. Finally, there is a conceptual risk of over-extending the synthesis (breadth) at the expense of depth, which we will manage by focusing and prioritising themes where explanatory value is highest for system change.

This study will make significant contributions to the field of realist research and to ongoing efforts to address health inequalities through systems change. Following participatory approaches, we involve diverse stakeholder groups, professionals, policymakers, researchers, students and citizens in all phases of the research in the four underlying projects, as well as in this overarching study. This ensures that dissemination is a continuous process rather than a final step, and that findings are closely aligned with practice. In co-creation with the intended target groups, we will develop tailored, accessible and actionable products. These products will include policy briefs, factsheets, transformative learning materials and a database of context-sensitive strategies for system change. The dissemination approach will be ongoing, dynamic and adaptive, guided by a living communication plan that aligns with ongoing developments and the communication channels of consortium partners. Activities will also include sharing milestones via social media, presenting at scientific and practitioner conferences, and developing targeted educational tools. Together, these efforts aim to foster awareness, learning and practical uptake of strategies to support system transformation for health equity.

The findings will be of value to professionals, policymakers and researchers seeking to understand and act on the structural and systemic drivers of health inequality, both within the Netherlands and internationally.
