# Supplementary material for: Exploring what works, for whom, under what circumstances to transform systems: realist synthesis protocol of four ongoing studies and literature addressing health inequalities
Source: BMJ Open. 2025 Dec 25;15(12):e110455. doi: 10.1136/bmjopen-2025-110455 (PMC12742087; doi:10.1136/bmjopen-2025-110455)
Supplement: Supplementary file 1 [file bmjopen-2025-110455.reviewer_comments.pdf]

PEER REVIEW HISTORY

BMJ Open publishes all reviews undertaken for accepted manuscripts. Reviewers are asked to complete a checklist review form and are provided with free text boxes to elaborate on their assessment. These free text comments are reproduced below.

ARTICLE DETAILS

Title (Provisional)

Exploring what works, for whom, under what circumstances to transform systems: realist synthesis protocol of four ongoing studies and literature addressing health inequalities

Authors

Horck, Stijn S.; Crone, Matty; Kamphuis, Carlijn B; Stevens, Gonneke; Dedding, Christine; Bussemaker, Jet; Van Der Pas, Suzan; Van Berkel, Jantien

---

VERSION 1 - REVIEW

**Reviewer**	**1**
**Name**	**Lahariya, Chandrakant**
**Affiliation**	**G. R. Medical College, Community Medicine**
**Date**	**01-Oct-2025**
**COI**	**None**

Study Objective & Research Questions

• There is some overlap between Questions 2 and 3, both of which concern mechanisms and strategies. Merging or refining these would sharpen the focus.

Methods & Analysis

o Critical Friends recruitment: The concept is innovative, but the selection process is insufficiently detailed. Please specify recruitment criteria, diversity considerations, and safeguards against tokenism.

o Literature search strategy: While iterative searching is consistent with realist reviews, greater transparency is needed. Please clarify inclusion/exclusion criteria, appraisal methods for grey literature, and how non-English publications will be handled.

o Dependence on underlying projects: The success of the synthesis is highly contingent on the progress of the four individual projects. Contingency plans for delays, data gaps, or divergent methodologies should be outlined.

o Terminology: Although Table 2 is helpful, terms like programme theory and CMO configuration should be briefly explained in the main text for accessibility to a broader readership.

Discussion

• The discussion highlights participatory strengths and the potential contributions of the project.

• However, it currently reads as descriptive rather than critically reflective. It would benefit from acknowledging and addressing key risks, such as:

o Potential conflicts or inconsistencies between findings across the four projects.

o The danger of overgeneralizing context-specific insights.

o Challenges in balancing breadth of synthesis with depth of analysis.

• By explicitly discussing these limitations and how you plan to mitigate them, the protocol will appear more rigorous and credible.

References:

Additional references to be considered:

1. Krishnan, A. (2025). Understanding Framing of Public Health Issues and Its Relevance in Climate Change Policy Discourse. Preventive Medicine Research & Reviews, 2: 99–101. DOI: 10.4103/PMRR.PMRR_257_24

2. Kumar, R. (2024). Health equity monitoring to achieve social justice: A preventive medicine perspective. Preventive Medicine Research & Reviews, 1: 162–163. DOI: 10.4103/PMRR.PMRR_60_23

Both references directly reinforce the health systems and equity focus of your article. Krishnan (2025) highlights how policy framing shapes public health discourse, which complements your paper's exploration of systems transformation and context–mechanism–outcome (CMO) dynamics. Including this work would situate your study within broader debates on policy discourse and complexity. Similarly, Kumar (2024) emphasizes the importance of equity monitoring for social justice, which aligns with your paper's goal of addressing health inequalities through realist synthesis. Adding these references will not only strengthen your theoretical grounding but also demonstrate engagement with recent regional scholarship in preventive medicine and public health, making the article more globally relevant and contextually robust.

**Reviewer**	**2**
**Name**	**Hoverd, Eleanor**

Affiliation **University of Oxford, Nuffield Department of Primary Care Health Sciences**

Date **25-Nov-2025**

COI **None**

Title : Exploring what works, for whom, under what circumstances to transform systems: realist synthesis protocol of four ongoing studies and literature addressing health inequalities

Thank you very much for inviting me to review this protocol that uses the realist approach in an innovative way. I am very interested to see how the findings turn out. I have made some minor suggestions below , mostly are grammatical . One point I have asked for clarity around is around theoretical saturation with that not being a typical goal of a realist approach. The authors may wish to revise this or explain their reasoning behind this aim. The figure is very helpful to the reader in understanding how this synthesis aims to work and I look forward to reading the results.

Step 1 Minor typo , the word “the” is missing between formed and basis : Based on these insights, a preliminary codebook will be developed, that will be used to analyse the literature that formed basis of the theoretical and empirical foundations of each project proposal.

43-45 Minor grammar : use future tense in this sentence given this is a protocol : For clarity, the themes are organised into groups and sub-groups to support further discussion and evaluation.

Step 2 Line 30 minor grammar : future tense needed in this sentence ? we will : This way, we systematically examine and reflect on the interactions between context, mechanism, and the intended outcome per theme.

Lines 45-49 Realist approaches tend not to aim for theoretical saturation as you are aiming to build, refine and test programme theory - seeking causal insights so you may want to consider how this sentence is phrased . If you are indeed seeking theoretical saturation you may want to explain how your approach differs to a realist one that does not seek thematic saturation but seeks to build causal insight and explanation about an intervention, programme or phenomenon

Step 3

Line 40 – just needs to be made future tense

Lines 47-50 can you provide a bit more clarity about who is in the heterogenous groups and homogenous groups? Or an example of who might be?

Step 4 Line 17-18 check tense

VERSION 1 - AUTHOR RESPONSE

Reviewer 1

We thank Reviewer 1 for the insightful and constructive comments, which have strengthened the clarity, methodological transparency, and rigour of the protocol. Below, we address each point in turn.

- ***There is some overlap between Questions 2 and 3, both of which concern mechanisms and strategies. Merging or refining these would sharpen the focus. Methods & Analysis***

Response:

We thank the reviewer for this valuable observation. To sharpen the distinction between the two questions, we have rephrased them in the Methods and analysis section (p. 7). The revised wording clarifies that question 2 focuses on identifying and understanding the mechanisms within the system (specifically how and why these mechanisms sustain or reduce health inequalities), while question 3 focuses on strategies and actions that can influence, activate, or disrupt these mechanisms. The revised questions now read:

2) Within these themes, what mechanisms are identified that explain how and why health inequalities are sustained or reduced?

3) What strategies and actions can influence, activate or disrupt these mechanisms to reduce health inequalities?

- ***Critical Friends recruitment: The concept is innovative, but the selection process is insufficiently detailed. Please specify recruitment criteria, diversity considerations, and safeguards against tokenism.***

Response:

We thank the reviewer for highlighting the need for greater clarity regarding the recruitment of Critical Friends. We have expanded this section in Step 3 section (p. 11) to specify recruitment criteria and diversity considerations. The following text has been added:

"In addition to these criteria, we will purposively recruit individuals with varied professional backgrounds, lived experiences of inequity, policy-level influence, and system-level implementation expertise. Diversity will be actively sought across dimensions such as sector, role, and social identity where relevant to the topic."

Furthermore, in the subsequent paragraph (also p. 11), we have clarified the composition of homogeneous and heterogeneous groups. This addition responds both to Reviewer 1's concerns about tokenism and Reviewer 2's request for clarification:

"Homogeneous groups will typically consist of participants with similar lived-experience backgrounds, whereas heterogeneous groups will consist of combinations of policymakers, practitioners, and researchers to support cross-perspective reflection. This approach ensures

that different types of knowledge can contribute on equal footing, reducing the risk that professional expertise overshadows lived-experience expertise during co-reflection and decision-making. It creates structured opportunities for all participants to shape the development, interpretation, and prioritisation of mechanisms and strategies, and helps prevent tokenistic involvement."

- ***Literature search strategy: While iterative searching is consistent with realist reviews, greater transparency is needed. Please clarify inclusion/exclusion criteria, appraisal methods for grey literature, and how non-English publications will be handled.***

Response:

We appreciate the reviewer's point and have expanded the description of our literature search strategy to provide greater transparency and alignment with realist review principles. Additional clarifications were added in two locations within Step 2. First, on p. 9, we elaborated on inclusion and exclusion criteria for scientific literature used for the assessment of the Initial Programme Theory (IPT). The following sentence has been added:

"Once a theme has been prioritised, studies will be included when they offer explanatory insight into how contexts, mechanisms, or strategies within that theme influence outcomes relevant to the IPT. Sources that only provide descriptive or correlational findings without explanatory contribution will be excluded."

Second, on p. 10, we expanded our description of grey literature appraisal and the use of non-English sources:

"We will purposively include grey literature that contributes to developing, refining, or testing the Initial Programme Theory (IPT), particularly when it provides insights into how contexts, mechanisms, or strategies operate that are not available in formal academic publications. While we will consider the methodological transparency and credibility of the sources, the primary criterion will be conceptual relevance for realist theory-building. As the consortium specifically investigates how Dutch systems maintain or reduce inequality, the relevant grey literature is predominantly Dutch and will therefore be included where conceptually relevant. As all consortium members are Dutch-speaking, these sources can be appraised without the need for translation.

- ***Dependence on underlying projects: The success of the synthesis is highly contingent on the progress of the four individual projects. Contingency plans for delays, data gaps, or divergent methodologies should be outlined.***

Response:

We thank the reviewer for raising this important point. Although the comment relates to the Methods and analysis section, we felt it was most appropriately addressed within the Discussion, where we reflect on the strengths and limitations of the overarching synthesis. We acknowledge that the synthesis is interdependent with the progress and methodological heterogeneity of the four underlying projects; however, the overarching project was intentionally designed to accommodate such variation. The realist synthesis does not rely on

uniform datasets but instead draws on patterns across multiple sources and iteratively tests theoretical propositions. This flexibility allows the overarching project to continue advancing even when individual projects progress at different speeds or produce uneven data. We have made this explicit in the Discussion (p. 13) with the following addition:

"However, certain limitations should be acknowledged. The synthesis depends on the progress, data availability and quality of the four underlying projects, which may differ in timing, methodological approaches, and contextual focus. To mitigate this, we will supplement insights from the projects with grey literature, stakeholder consultation, and iterative CMO testing across alternative sources where needed."

- ***Terminology: Although Table 2 is helpful, terms like programme theory and CMO configuration should be briefly explained in the main text for accessibility to a broader readership.***

Response:

We appreciate this suggestion to enhance readability for a broader audience. In response, we have added concise in-text explanations of key realist terms at the points where they are first introduced. Specifically: Initial Programme Theory (IPT): In Step 1 (p. 8) we now clarify its meaning by adding:

"…to develop a rough Initial Programme Theory (IPT), which is a preliminary explanation of how change is expected to occur, through which mechanisms, and under what conditions."

Context–Mechanism–Outcome (CMO) configurations: In Step 2 (p. 9) we added:

"Once a key theme is identified for further investigation, the next step involves formulating Context–Mechanism–Outcome (CMO) configurations, which are the core analytical units in realist research and describe how particular contexts may trigger mechanisms that produce specific outcomes (Jagosh et al., 2012)."

- ***The discussion highlights participatory strengths and the potential contributions of the project. However, it currently reads as descriptive rather than critically reflective. It would benefit from acknowledging and addressing key risks, such as:***
    - ***Potential conflicts or inconsistencies between findings across the four projects.***
    - ***The danger of overgeneralizing context-specific insights.***
    - ***Challenges in balancing breadth of synthesis with depth of analysis.***

Response:

We thank the reviewer for this constructive suggestion. In response, we have substantially strengthened the Discussion section by adding a new paragraph that explicitly acknowledges key risks and limitations. The revised structure now separates the Discussion into two clear parts: (1) strengths and limitations of the study, and (2) considerations for dissemination. This enhances critical reflection and directly addresses the reviewer's concerns. The following paragraph has been added to the Discussion (p. 13):

"This protocol has notable strengths, including its prospective realist design, structured cross-project synthesis, and participatory involvement of stakeholders, which supports theory refinement across real-world settings. However, certain limitations should be acknowledged. The synthesis depends on the progress, data availability and quality of the four underlying projects, which may differ in timing, methodological approaches, and contextual focus. To mitigate this, we will supplement insights from the projects with grey literature, stakeholder consultation, and iterative CMO testing across alternative sources where needed. Additionally, insights emerging from specific contexts may not be directly transferable to other settings. To address this, the analysis will prioritise explanatory patterns rather than universal claims, highlighting contextual contingencies and boundary conditions. Finally, there is a conceptual risk of over-extending the synthesis (breadth) at the expense of depth, which we will manage by focusing and prioritising themes where explanatory value is highest for system change."

- ***Additional references to be considered***

Response:

We appreciate the reviewer's suggestion to include these additional references. Both papers strengthen the health equity and policy framing dimensions of our introduction, and we agree that they enhance the conceptual grounding of the protocol. We have therefore incorporated them into the Introduction (p. 5) as follows:

"From this perspective, health outcomes are seen not as the result of isolated individual choices, but as patterned behaviours shaped, ***framed*** *and constrained by broader systemic conditions (Krishnan, 2025)."*

"As a result, the structural barriers remain in place, hindering sustainable ***and equity-oriented*** *system improvements (Dijkstra et al., 2025; Kumar, 2024)."*

Reviewer 2

We thank Reviewer 2 for the encouraging feedback and helpful detailed suggestions, particularly regarding grammar, clarity, and alignment with realist methodology.

- ***One point I have asked for clarity around is around theoretical saturation with that not being a typical goal of a realist approach. The authors may wish to revise this or explain their reasoning behind this aim.***

Response:

We thank the reviewer for this insightful observation. We agree that our original phrasing unintentionally suggested an aim of theoretical saturation, which is indeed not consistent with realist approach. Our intention was instead to convey that data collection and theory refinement would continue until no new conceptual insights emerge for the specific CMO configurations being developed. This aligns with realist iterative refinement rather than

thematic or theoretical saturation. To clarify this, we have revised the text in Step 2 (p. 10) as follows:

"Unlike a systematic review, the aim is not exhaustiveness but to purposively identify evidence on the review themes that develops and refines CMO configurations, continuing until no new conceptual insights emerge."

- ***Grammar and tense corrections***

Response:

We thank the reviewer for their careful editorial attention. All suggested grammar and tense corrections have been incorporated into the revised manuscript and are visible in the tracked-changes version.

- ***Can you provide a bit more clarity about who is in the heterogenous groups and homogenous groups? Or an example of who might be?***

Response:

We thank the reviewer for this helpful request for clarification. To explain more clearly how group composition supports meaningful participation, we have expanded the description in the Methods and Analysis section (p. 9). The revised text now reads:

"Homogeneous groups will typically consist of participants with similar lived experience backgrounds, whereas heterogeneous groups will consist of combinations of policymakers, practitioners and researchers to support cross-perspective reflection. This approach ensures that different types of knowledge can contribute on equal footing, reducing the risk that professional expertise overshadows lived-experience expertise during co-reflection and decision-making. It creates structured opportunities for all participants to shape the development, interpretation, and prioritisation of mechanisms and strategies, and helps prevent tokenistic involvement."
